# De-Labeling of Penicillin Allergy in the Emergency Department at a Veterans Affairs Hospital

**DOI:** 10.5811/westjem.48957

**Published:** 2026-06-28

**Authors:** Brian Chen, Phuong Khanh T. Nguyen, My-Phuong Pham, Cynthia Koh, Christopher J. Graber

**Affiliations:** * Veterans Affairs Greater Los Angeles Healthcare System, Department of Pharmacy, Los Angeles, California; † Veterans Affairs Greater Los Angeles Healthcare System, Department of Emergency Medicine, Los Angeles, California; ‡ Veterans Affairs Greater Los Angeles Healthcare System, Department of Infectious Disease, Los Angeles, California

## Abstract

**Introduction:**

Penicillin allergy is one of the most reported drug allergies, but studies have shown that patients who report an allergy frequently do not have a significant reaction when challenged. Avoidance of penicillin or beta-lactam antibiotics in these patients results in increased hospitalization costs, suboptimal antibiotic therapy, and risk for subsequent infection with multidrug resistant organisms.

**Methods:**

This was a single-center, retrospective chart review that included patients ≥ 18 years of age with documented penicillin allergy who presented to the emergency department (ED) over a one-year period. Clinical pharmacy specialists in the ED reviewed and stratified patient allergy risk. Patients with low-risk allergy histories who were to be admitted and gave formal consent were given an oral amoxicillin challenge and then observed in the ED for at least one hour. Patients who were eligible for challenge but were to be discharged from the ED were given the option to be seen in the allergy clinic. The primary outcome was the incidence of adverse reactions related to the oral amoxicillin challenge for patients. An exploratory secondary outcome was the average length of stay (LOS) in the ED for oral amoxicillin-challenged patients compared to the average LOS for non-oral amoxicillin-challenged patients. We performed descriptive statistics on all variables.

**Results:**

A total of 144 patients received an allergy assessment in the ED, 20 who received an oral amoxicillin challenge and 124 who did not. Baseline characteristics were similar in both groups. The average total LOS in the oral amoxicillin-challenged patients was a non-significant 36 minutes longer compared to the non-challenged patients (P = 0.4). Of 144 patients, 71 (49%) had their penicillin allergy removed as a result of the challenge.. One patient experienced a mild and self-limited reaction to the oral amoxicillin challenge (0.7%; 95% CI, 0.02–3.8%).

**Conclusion:**

An ED pharmacist-led, penicillin de-labeling protocol was safe and feasible. A secondary exploratory analysis showed no significant difference in length of stay between patients who received the oral amoxicillin challenge in the ED vs those who did not. Performing a proper penicillin allergy evaluation in the ED can have lasting benefits for patients and antimicrobial stewardship.

## INTRODUCTION

Penicillin allergy is a commonly reported drug reaction. Although it is widely reported, about 80–95% of individuals with self-reported penicillin allergy histories can tolerate penicillin after a proper allergy assessment.^1^–^4^ Efforts to de-label penicillin allergies have extended even to the emergency department (ED) with success.^5^–^6^ Clinically significant immunoglobulin E (Ig-E)-mediated type I hypersensitivity is uncommon and rare, reported as < 5%.^7^ Despite the frequency of reported penicillin allergies, about 90% of patients have been shown to tolerate beta-lactams, as the cross-reactivity between penicillins and cephalosporins is relatively low at 1–3%.^8^–^11^ This is likely due to variations in the side chains attached to the core beta-lactam ring being the major determinants of allergic cross-reactivity, rather than the beta-lactam ring itself.^12^ Furthermore, approximately 80% of patients with IgE-mediated penicillin allergy will lose their sensitivity after 10 years.^13^ In a systematic review and meta-analysis, in a cohort of > 5,000 participants who had reported penicillin allergy and underwent drug challenge, ≥ 94% tolerated the penicillin challenge without any significant reaction.^4^

Inappropriate and unverified antibiotic-allergy documentation in patients’ health records can be recorded incorrectly after a non-immunological reaction such as gastrointestinal (GI) upset, headache, or fatigue.^2^ Some patients may be exposed to unnecessary extended-spectrum antibiotics given the self-reported penicillin allergy. Studies show that most patients who report penicillin allergy are unnecessarily avoiding penicillin-class antibiotics because either their penicillin allergy waned over time or previous reactions should not have been attributed to penicillin.^1^,^13^ Penicillin allergy labels have a direct impact on antimicrobial stewardship by resulting in use of potentially less effective and unnecessarily broad-spectrum antibiotics.

Currently, many healthcare professionals in clinical practice will avoid using penicillins or b-lactam antibiotics in patients with a reported penicillin allergy. However, this significantly limits appropriate antibiotic choice, resulting in increasing hospitalization costs, suboptimal antibiotic therapy, risk for subsequent infections with multidrug resistant organisms (eg, methicillin-resistant *Staphylococcus aureus*, vancomycin-resistant *Enterococcus*, and *Clostridioides difficile*).^1^–^2^, ^15^–^17^ Common alternatives to penicillin include vancomycin, clindamycin, and fluoroquinolones, which have been associated with greater adverse effects than beta-lactams, including nephrotoxicity and risk for *C difficile* infection.^18^ Moreover, a recent meta-analysis revealed that patient outcomes worsen beyond infectious concerns, with a penicillin allergy alone being associated with increased mortality, as well as heart failure and increased intensive care unit and ventilation complications.^19^

Regardless of specific clinical harms, the impact of penicillin labels on operational outcomes has been demonstrated, with multiple studies demonstrating that a penicillin allergy label is associated with longer hospital length of stay (LOS).^20^–^22^ Therefore, appropriate penicillin allergy assessment and management should be an important component of antibiotic stewardship, which can significantly improve treatment outcomes, decrease healthcare costs, and prevent antibiotic resistance. Here we evaluate a penicillin allergy assessment effort implemented in a Veterans Affairs emergency department (ED).

**Population Health Research Capsule**

What do we already know about this issue?

*Most reported penicillin allergies are not true IgE-mediated allergies, and de-labeling can safely expand beta-lactam use*.

What was the research question?

*Can a pharmacist-led ED penicillin de-labeling protocol safely remove penicillin allergy labels? Does this affect ED length of stay (LOS)?*

What was the major finding of the study?

*60 patients out of 144 (42%) were de-labeled directly in the ED, with one mild reaction (0.7%, 95% CI 0.02–3.8%) that required no intervention*.

How does this improve population health?

*Removing inaccurate penicillin allergies in the ED supports safer, narrower antibiotic use and may have multiple clinical and operational benefits*.

## METHODS

This was a single-center, retrospective chart review of adult veterans ≥ 18 years of age with a penicillin allergy who presented to the ED at the Veterans Affairs Greater Los Angeles Healthcare System (VAGLAHS) from January 1–December 31, 2021. Charts were reviewed based on the inclusion of a note specific to the pharmacy workflow detailed in Figure 1. One author (BC) reviewed all charts using a standardized form, and this review was monitored periodically by two other authors (PKN, MP) to ensure uniformity. As informed by Worster et al,^23^ we defined case selection criteria and variables, used an abstraction form, monitored performance, discussed interobserver reliability, identified the medical record, and obtained institutional review board (IRB) approval. The study was approved by the IRB by its standard protocol, including obtaining formal consent from all participants. We processed data on secure servers in accordance with institutional policies. There were no missing data for the key variables. Using the VAGLAHS ED Antibiotic Stewardship Program Pharmacy Penicillin Allergy Assessment and Intervention Workflow (Figure), ED clinical pharmacy specialists (CPS) reviewed and stratified patient allergy risk.

Patients with history of penicillin intolerance (eg, an adverse effect such as GI upset, nausea, vomiting, or diarrhea that was not a true allergy) had the penicillin allergy de-labeled from their patient profile, as did those who denied an allergy and those who did not know what their allergy was. Patients with moderate-high and high-risk allergy histories were not de-labeled, nor were they challenged in the ED; however, they were provided the option to be further assessed at the Allergy/Immunology Outpatient Clinic.

Patients were challenged with a single dose of amoxicillin 250 mg by mouth if they 1) were determined to have a low-risk history, 2) formally consented to the challenge, and 3) carried a disposition for admission. These criteria were chosen to limit the risk of a severe adverse reaction, and in the case one occurred ensured that the patient would be able to be appropriately monitored and treated. The only patients who met these criteria but were not challenged were deemed by the care team to be too unstable. For patients who were eligible to receive the oral amoxicillin challenge but were to be discharged from the ED, they were given the option to be seen in the Allergy/Immunology Outpatient Clinic. Amoxicillin was the only antibiotic given in this challenge.

The treating ED team carried out observation for one hour after the dose was administered, with monitoring and intervention at the team’s discretion. Severity of the reaction was defined as mild if no intervention was needed; moderate if intervention was needed but required no upgrade in level of care; severe tier 1 if intermediate or intensive care was needed but no anaphylaxis was noted; and severe tier 2 if anaphylaxis was noted. The primary outcome was the incidence of adverse reactions related to the oral amoxicillin challenge for patients. An exploratory secondary outcome was the average LOS in the ED for oral amoxicillin-challenged patients compared to the average LOS for non-oral amoxicillin-challenged patients.

We performed descriptive statistics on all variables. We used the *t*-test to compare continuous data and chi-square test to compare categorical data. For all tests and analyses, statistical significance was determined with a *P* value < .05 (two-sided significance).

## RESULTS

Baseline characteristics of the studied population, including classification based on allergy severity, are shown in Table 1. The ED LOS times for challenged vs non-challenged patients are presented in Table 2.

Of 144 patients who received penicillin allergy assessment in the ED via the protocol outlined in Figure 1, 71 (49%) were ultimately able to have their penicillin allergy de-labeled. Furthermore, 60 patients (42%) were de-labeled directly in the ED—19 (32%) through pharmacist assessment followed by penicillin challenge, and 41 (68%) via pharmacist assessment alone. An additional 11 patients (15%) were de-labeled after allergy clinic follow-up. Of the 20/144 patients (14%) who received the oral amoxicillin challenge, only one (0.7%; 95% CI, 0.02–3.8%) had a mild reaction that required no specific intervention, and his allergy was not de-labeled.

Of 58 patients referred to the allergy clinic, 24 (41%) presented for their appointment, and among those 24 patients 11 (46%) had their labels removed; the rest were lost to follow-up. The average total LOS in the oral amoxicillin-challenged patients was 401.25 (SD 121.99) minutes vs 365.73 (171.99) minutes in the non-challenged patients (*P* = .4) (Table 2). The majority of patients in both groups had their last penicillin allergy > 10 years prior, and/or it had occurred when they were < 18 years of age. Therefore, this study supports the hypothesis that patients may lose their hypersensitivity to penicillin after 10 years. A dermatologic reaction was the most common allergy reaction reported in both groups. Finally, a significant benefit noted was that of the patients challenged, over half of them went on to safely receive treatment with a beta-lactam (Table 3).

## DISCUSSION

Our ED-pharmacist-led, penicillin de-labeling study is safe and feasible for any ED with a dedicated pharmacist; our study led to challenged patients safely receiving subsequent beta-lactam therapy. As a strictly exploratory measure, there was no significant difference in average ED total LOS between patients who received the oral amoxicillin challenge compared to those who did not receive the challenge. Consideration of LOS, while important, should also be de-emphasized in view of the broader positive impact on the overall morbidity and mortality of penicillin allergy-labeled patients, which has been suggested by previous research and must be considered as potentially more impactful than operational concerns.

Removing mislabeled penicillin allergy has far-reaching downstream effects for improved patient outcomes. By removing inappropriate and unverified penicillin allergy labeling for any future hospitalizations, patients will receive appropriate empiric antibiotic therapy, if clinically indicated. This would decrease the risk for multidrug resistant organisms and *C. difficile* infection, potentially lowering healthcare costs and improving multiple patient outcomes beyond antibiotic stewardship alone. The ED is a valuable place to intervene, and this research demonstrates that it can be operationalized safely, with a suggestion that it can be done without significant impact to ED workflow.

## LIMITATIONS

An inherent bias of our protocol was the decision to challenge only those with low-risk allergy histories who were then admitted, with the rationale that this would be least likely to impact LOS. This study subsequently cannot provide a comparator group for challenged patients who were then discharged. However, the suggestion that the addition of an amoxicillin challenge did not appear to add to ED operational cost more than a typical admitted patient is a powerful argument in favor of the feasibility of this protocol, and against those who may be skeptical of the potential operational impact of a de-labeling effort. Additionally, there were patients who were eligible for the penicillin allergy assessment but were unable to undergo the oral amoxicillin challenge due to the clinical acuity of their presentation. These patients had low-risk allergy histories, but because of their clinical needs they were not administered the challenge to mitigate any additional risk associated with it.

Secondly, there were patients who declined to receive the oral amoxicillin challenge and/or outpatient allergy clinic consult. These patients elected to keep their penicillin allergy on their profile without any further workup. Lastly, as this study was done during the COVID-19 pandemic, it was noted that fewer allergy assessments were conducted during pandemic surges, as the ED faced larger patient volumes with higher acuity, higher risk patients. An additional consideration is the setting of our ED within a Veterans’ Affairs hospital, which may carry its own operational features that may differ from others. However, our protocol’s main operational requirements (other than typical ED operations) are an ED pharmacist and an outpatient allergy referral, with no particularly unusual measures to engage either. Therefore, we feel that this protocol will be highly reproducible in any ED with these resources.

The use of the allergy assessment protocol has expanded within this VA hospital to include inpatient services. Possible future directions include evaluating the rate of penicillin allergy re-labeling; for example, in this study, there was one patient who was excluded due to having their penicillin allergy removed, with subsequent re-labeling at a later ED visit. Therefore, future studies can assess how often penicillin allergy re-labeling occurs and the safety outcomes associated with initial penicillin allergy de-labeling. Furthermore, this study can be expanded to beta-lactam class allergy evaluation as there is low cross-hypersensitivity between the penicillin and beta-lactam classes.

## CONCLUSION

There was no significant difference in the average ED total length of stay for patients who received an oral amoxicillin challenge vs those who did not receive the challenge. The impact of penicillin allergy assessment and appropriate de-labeling has far-reaching benefits for patients and antimicrobial stewardship. A proper evaluation, including assessing prior tolerance history, and subsequent documentation or de-labeling of penicillin allergy, are crucial to providing patients with optimal therapy for their suspected infection.

## Figures and Tables

**Figure:**
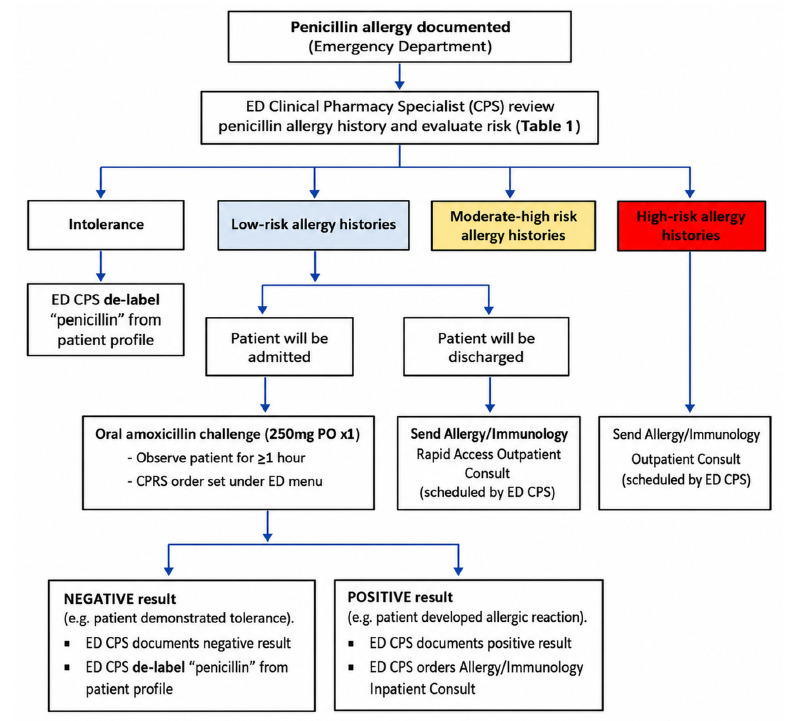
Pharmacist workflow in a study describing an emergency department-based, pharmacist-led penicillin allergy de-labeling protocol. *CPRS*, computerized patient record syste**m**; *ED*, emergency department.

**Table 1:** Characteristics of patient population in a study examining outcomes of an emergency department-based, pharmacist-led protocol to de-label penicillin allergy.

Characteristic	Received oral amoxicillin challenge (n = 20)	Did not receive oral amoxicillin challenge (n = 124)	Total (N = 144)
Age – years, average	69 (SD 12.6)	66.4 (SD 15.7)	66.8 (SD 15.3)
Male sex – n (%)	18 (90)	114 (92)	132 (92)
Race – n (%)			
White	14 (70)	57 (46)	71 (49)
Black	5 (25)	48 (39)	53 (37)
Hispanic	0 (0)	1 (1)	1 (1)
Other	0 (0)	6 (5)	6 (4)
Declined to answer	1 (5)	12 (9)	13 (9)
Drug allergies – n (%)			
Penicillin	4 (20)	47 (38)	51 (35)
Beta-lactams	1 (5)	2 (2)	3 (2)
More than 1 class of allergies, including antibiotics	15 (75)	75 (60)	90 (63)
Comorbidities			
Charlson Comorbidity Index score (average)	5.5 (SD 3)	4.9 (SD 3.35)	4.9 (SD 3.3)
Emergency Severity Index – n (%)			
1 – immediate, life-saving intervention required	0 (0)	0 (0)	0 (0)
2 – high risk of deterioration	2 (10)	9 (7)	11 (8)
3 – stable with multiple types of resources needed to investigate or treat	16 (80)	98 (79)	114 (79)
4 – stable with only one type of resource anticipated	2 (10)	17 (14)	19 (13)
5 – stable with no resources needed except oral or topical medications	0 (0)	0 (0)	0 (0)
Penicillin allergy history – n (%)			
Date of last PCN allergy			
> 10 years	20 (100)	110 (89)	130 (90)
< 10 years	0 (0)	14 (11)	14 (10)
Age of last allergic reaction			
< 18 years old	10 (50)	44 (36)	54 (38)
> 18 years old	6 (30)	39 (31)	45 (31)
Unknown	4 (20)	41 (33)	45 (31)
Type of last penicillin allergic reaction – n (%)			
Dermatologic	9 (45)	45 (36)	54 (38)
Gastrointestinal (N/V/D)	0 (0)	9 (7)	9 (6)
Other intolerances / hypersensitivity (type 2,3,4)	4 (20)	22 (18)	26 (18)
Anaphylaxis	0 (0)	15 (12)	15 (10)
Denies allergy	1 (5)	12 (10)	13 (9)
Unknown	6 (30)	21 (17)	27 (19)
Severity of penicillin allergy history – n (%)			
Intolerance	0 (0)	13 (10)	13 (9)
Low	15 (75)	44 (36)	59 (41)
Moderate	5 (25)	38 (31)	43 (30)
High	0 (0)	29 (23)	29 (20)

*N/V/D*, nausea, vomiting, diarrhea; *PCN*, penicillin; *SD*, standard deviation.

**Table 2:** Results of a study examining outcomes of an emergency department-based, pharmacist-led penicillin allergy de-labeling protocol.

Outcome	Patients who received oral amoxicillin challenge (n = 20)	Patients who did not receive oral amoxicillin challenge (n = 124)	Total patients (N = 144)	*P* value
ED total length of stay – minutes (average) (SD)				
	401.3 (122)	365.7 (172)	370.7 (166)	.4
Penicillin allergy removed in the ED? – n (%)				
Yes	19 (95)	41 (33)	60 (42)	
No	1 (5)	83 (67)	84 (58)	
Allergy consult placed? – n (%)				
Yes	0 (0)	58 (47)	58 (40)	
No	20 (100)	66 (53)	86 (60)	

*ED*, emergency department; *SD*, standard deviation.

**Table 3:** Subsequent use of beta-lactams in 20 patients who had received an oral amoxicillin challenge in the emergency department through a pharmacist-led penicillin allergy de-labeling protocol.

n (%)	
Penicillin	7 (35)
Cephalosporins	4 (20)
Carbapenems	0 (0)
More than 1 class of beta-lactams	1 (5)

## References

[1] Pongdee T (2018). Evaluation and management of penicillin allergy. Mayo Clinic Proceedings.

[2] Blumenthal KG, Peter JG, Trubiano JA (2019). Antibiotic allergy. Lancet.

[3] Bigby M, Jick S, Jick H (1986). Drug-induced cutaneous reactions. a report from the Boston Collaborative Drug Surveillance Program on 15,438 consecutive inpatients, 1975 to 1982. JAMA.

[4] DesBiens M, Scalia P, Ravikumar S (2020). A closer look at penicillin allergy history: systematic review and meta-analysis of tolerance to drug challenge. Am J Med.

[5] Raja AS, Lindsell CJ, Bernstein JA (2009). The use of penicillin skin testing to assess the prevalence of penicillin allergy in an emergency department setting. Ann Emerg Med.

[6] Marwood J, Aguirrebarrena G, Kerr S (2017). De-labelling self-reported penicillin allergy within the emergency department through the use of skin tests and oral drug provocation testing. Emerg Med Australas.

[7] Shenoy ES, Macy E, Rowe T (2019). Evaluation and management of penicillin allergy: a review. JAMA.

[8] Campbell S, Hauler G, Immler EL (2020). Pharmacist-led penicillin allergy assessment in the emergency department reduced empiric fluoroquinolone use. Clin Infect Dis.

[9] Blumenthal KG, Wickner PG, Hurwitz S (2017). Tackling inpatient penicillin allergies: assessing tools for antimicrobial stewardship. J Allergy Clin Immunol.

[10] Campagna JD, Bond MC, Schabelman E (2012). The use of cephalosporins in penicillin-allergic patients: a literature review. J Emerg Med.

[11] MacFadden DR, LaDelfa A, Leen J (2016). Impact of reported beta-lactam allergy on inpatient outcomes: a multicenter prospective cohort study. Clin Infect Dis.

[12] Turner J, Muraoka A, Bedenbaugh M (2022). The chemical relationship among beta-lactam antibiotics and potential impacts on reactivity and decomposition. Front Microbiol.

[13] Joint Task Force on Practice Parameters; American Academy of Allergy, Asthma and Immunology; American College of Allergy, Asthma and Immunology; Joint Council of Allergy, Asthma and Immunology (2010). Drug allergy: an updated practice parameter. Ann Allergy Asthma Immunol.

[14] Thompson DF, Ramos CL (2017). Antibiotic-induced rash in patients with infectious mononucleosis. Ann Pharmacother.

[15] Miguel A, Azevedo LF, Araujo M (2012). Frequency of adverse drug reactions in hospitalized patients: a systematic review and meta-analysis. Pharmacoepidemiol Drug Saf.

[16] Blumenthal KG, Lai KH, Huang M (2017). Adverse and hypersensitivity reactions to prescription nonsteroidal anti-inflammatory agents in a large health care system. J Allergy Clin Immunol Pract.

[17] Stone CA, Trubiano J, Coleman DT (2020). The challenge of de-labeling penicillin allergy. Allergy.

[18] Blumenthal K, Lu N, Zhang Y (2018). Risk of meticillin resistant *Staphylococcus aureus* and *Clostridium difficile* in patients with a documented penicillin allergy: population based matched cohort study. BMJ.

[19] Zhang S, Dong T, Xian J (2025). Association between penicillin allergy labels and serious adverse events in hospitalized patients: a systematic review and meta-analysis. Front Pharmacol.

[20] Sousa-Pinto B, Cardoso-Fernandes A, Araújo L (2018). Clinical and economic burden of hospitalizations with registration of penicillin allergy. Ann Allergy Asthma Immunol.

[21] Ali SB, Hughes T, Smith A (2024). Penicillin or cephalosporin antibiotic allergy label: Influence on length of stay and hospital readmission. J Allergy Clin Immunol Glob.

[22] Huang KG, Cluzet V, Hamilton K (2018). The impact of reported beta-lactam allergy in hospitalized patients with hematologic malignancies requiring antibiotics. Clin Infect Dis.

[23] Worster A, Bledsoe RD, Cleve P (2005). Reassessing the methods of medical record review studies in emergency medicine research. Ann Emerg Med.

